# Hyperspectral imaging for pest symptom detection in bell pepper

**DOI:** 10.1186/s13007-024-01273-5

**Published:** 2024-10-03

**Authors:** Marvin Krüger, Thomas Zemanek, Dominik Wuttke, Maximilian Dinkel, Albrecht Serfling, Elias Böckmann

**Affiliations:** 1 Julius Kühn-Institute, Federal Research Center for Cultivated Plants, Institute for Plant Protection in Horticulture and Urban Green, Braunschweig, Germany; 2 Julius Kühn-Institute, Federal Research Center for Cultivated Plants, Institute for Resistance Research and Stress Tolerance, Quedlinburg, Germany; 3 Wolution GmbH & Co. KG, Planegg, Germany

**Keywords:** Monitoring, Pest detection, Noninvasive, Automated, Robot

## Abstract

**Background:**

The automation of pest monitoring is highly important for enhancing integrated pest management in practice. In this context, advanced technologies are becoming increasingly explored. Hyperspectral imaging (HSI) is a technique that has been used frequently in recent years in the context of natural science, and the successful detection of several fungal diseases and some pests has been reported. Various automated measures and image analysis methods offer great potential for enhancing monitoring in practice.

**Results:**

In this study, the use of hyperspectral imaging over a wide spectrum from 400 to 2500 nm is investigated for noninvasive identification and the distinction of healthy plants and plants infested with *Myzus persicae* (Sulzer) and *Frankliniella occidentalis* (Pergande) on bell peppers. Pest infestations were carried out in netted areas, and images of single plants and dissected leaves were used to train the decision algorithm. Additionally, a specially modified spraying robot was converted into an autonomous platform used to carry the hyperspectral imaging system to take images under greenhouse conditions. The algorithm was developed via the XGBoost framework with gradient-boosted trees. Signals from specific wavelengths were found to be associated with the damage patterns of different insects. Under confined conditions, *M. persicae* and *F. occidentalis* infestations were distinguished from each other and from the uninfested control for single leaves. Differentiation was still possible when small whole plants were used. However, application under greenhouse conditions did not result in a good fit compared to the results of manual monitoring.

**Conclusion:**

Hyperspectral images can be used to distinguish sucking pests on bell peppers on the basis of single leaves and intact potted bell pepper plants under controlled conditions. Wavelength reduction methods offer options for multispectral camera usage in high-grown vegetable greenhouses. The application of automated platforms similar to the one tested in this study could be possible, but for successful pest detection under greenhouse conditions, algorithms should be further developed fully considering real-world conditions.

## Background

Integrated pest management (IPM) is globally endorsed as a paradigm for crop protection. In practice, several pest management elements are combined, and chemical treatments are carried out only when nonchemical measures fail to control pest organisms. This failure is detected by monitoring and comparing the results to damage thresholds. Early and automated detection of plant damage is becoming an important part of this decision-making process [1] because it can help reduce the overall workload and costs, as well as the use of chemical products for plant protection.

In this context, advanced technologies are becoming increasingly explored. Hyperspectral imaging (HSI) is a technique that has been used frequently in recent years in the context of natural science, and the successful detection of several fungal diseases [2, 3] and some pests [4, 5] has been reported. In this study, bell pepper was selected as a model crop because of its smooth leaf structure, slow growth and uncomplicated culture to explore the potential of pest and pest symptom detection via HSI for vegetables. Additionally, infestations of two economically relevant pests that affect bell peppers, *Myzus persicae* and *Frankliniella occidentalis*, were distinguished from each other and from noninfested plants. For both pests, direct monitoring of adults via sticky traps is common, and there are several approaches used to identify these insects on traps or plants [6], such as HSI [7]. When traps are used for early detection, regular monitoring of plants is needed to estimate population densities, especially for aphids [8]. Therefore, regular, direct and nondestructive measurements of plants that include spatial information are the best choice for monitoring.

In this context, HSI can be used to automate the detection of pest damage on plants and to assess damage unobservable via manual methods, such as systemic changes in plants [9]. Notably, HSI is an important technique in remote sensing, and the electromagnetic spectrum from the visible to the near-infrared wavelength ranges is obtained. Combining the main advantages of spectroscopy and computer vision, HSI methods can simultaneously acquire spectral and spatial information in one system. HSI classification, i.e., assigning each pixel to a certain class on the basis of its spectral characteristics, is a popular research task in the hyperspectral community and has drawn broad attention in the remote sensing field. In HSI classification tasks, two main challenges exist: (1) the large spatial variability of spectral signatures and (2) the limited availability of training samples versus the high dimensionality of hyperspectral data [10]. In the current study, algorithms based on machine learning are used for the evaluation of hyperspectral images. These algorithms automatically learn to recognize pest symptoms from training datasets.

Training datasets for the bell pepper model and the pests *F. occidentalis* and *M. persicae* were generated from single-leaf images considering the corresponding pest symptoms. The accuracy of the resulting algorithm was assessed based on its ability to distinguish the two pests from each other and from a pest-free control with leaf images from test datasets. The most significant wavelengths for *M. persicae* and *F. occidentalis* detection were subsequently identified. Furthermore, the recognition accuracy of the developed algorithm was tested on potted bell pepper plants under controlled conditions and plants cultivated under practical conditions in a greenhouse.

## Methods

### Controlled condition experiment

#### General setup

Bell pepper plants of Bedingo F1 (Rijk Zwaan Welver GmbH, Welver, Germany) were sown in 5 batches from May to August 2021. After sowing, the young plants were germinated on substrate from Klasmann-Deilmann (Klasmann-Deilmann GmbH, Geeste, Germany) in 600 × 400 × 60 mm plant bowls and were separated after 4 weeks into 2 l pots 150 mm in diameter.

The plants were maintained at a constant temperature of 23 ± 1.5 °C and 50–70% relative humidity on grid tables in greenhouse chambers. The plants were then transferred to insectary cages with dimensions of 60 × 60 × 120 mm and a 160 μm mesh size (Bugdorm-2120 F, MegaView Science, Taichung, Taiwan). The cages were randomly placed on the tables. The plants were grown for 4 weeks at a constant 22 °C and 20% humidity, with a shading limit of 400 W.

Sixteen plants from each batch were used per pest treatment. These plants were inoculated with *Myzus persicae* [MP] or *Frankliniella occidentalis* [FO]. Additionally, 13 or 14 plants per batch were kept pest free as controls [UC]. The higher number of plants in pest treatments was taken to account for non-establishment of pests. Higher numbers of plants in the pest treatments were used to balance cases where pest infestation may fail. If no pest was established, the plants were not subjected to image acquisition. Insects were reared on peppers at 25 °C with a 16/8 day/night cycle. Inoculation was carried out with 10 adult *Myzus persicae* individuals and 20 adult *Frankliniella occidentalis* individuals two weeks before measurement via HSI. Insects were transferred to leaves in the upper third of each plant. All the plants were hand watered on average every 48 h, and the amount of water for the plants was adjusted according to the soil moisture level needed for healthy plant growth in each pot. At the time of HSI measurement, the bell pepper plants averaged 550 mm ± 150 mm in height.

#### Hyperspectral image measurements under controlled conditions

For image acquisition, a dual camera setup consisting of a HySpex VNIR-1800 and a HySpex SWIR-384me was used. The cameras recorded files with the following specifications: VNIR-1800, SN-0824 = 404–994 nm, 184 bands, resolution of 1800 × 4500, spectral resolution of 3.5 nm, and − 3.5 GB per image; SWIR-384me, SN-3122 = 954–2511 nm, 288 bands, resolution of 384 × 988, spectral resolution of 7 nm, and a file size of approximately 150–250 MB per image. The imaging of the cage-grown peppers with the hyperspectral cameras was carried out at the “PhenoScan” measuring platform of the Julius Kühn Institute. PhenoScan is an opaque chamber in which the sample material is positioned on a reflectionless base. A linear motor moves the camera along a defined line at an adjustable distance. In this experimental setup, the camera was 800 mm above the plants or leaves that were placed horizontally below the camera. A 1 m-focus lens was used to obtain the images. An actively cooled studio lamp from Hedler (Runkel, Germany) was used for illumination. The illuminant used was an Osram HLX 400 W (Munich, Germany). The lamp was screwed next to the camera and moved with it. A beryllium white plate served as a reflection reference for the camera and was included in each image.

Using this setup, first, an image of each individual plant was taken, with the plant placed horizontally under the camera. Then, all the leaves of an individual plant were dissected and arranged in one image with the top sides facing upward, and a second image with the bottom side of the leaves facing upward was taken. Some images were noisy or not saved correctly, reducing the number of available images; only image pairs that were available from both cameras and for both sides of leaves were used for further analysis. Table 1 shows the number of plants reared and the resulting number of available images.

**Table 1:** Distribution of the available image files

Class	Number of plants	Total number of leaves	Images of plants	Images of leaves per side
Control group	66	772	66	64
*Myzus persicae*	80	902	73	73
*Frankliniella occidentalis*	80	887	63	64

The number of plants grown in the treatments, the number of leaves on these plants, the number of images (with one plant per image) and the number of paired images of leaves (both side) are listed. All leaf images were taken from the top and bottom sides. All the leaf and plant images were obtained along the same line via the VNIR and the SWIR cameras.

#### Pest monitoring under controlled conditions

Additionally to the HSI process, the pests and pest damage were evaluated. For this purpose, the pest numbers and typical symptoms in terms of percentage of leaf area damaged were recorded for each leaf from all plants per treatment. Nymphs and adults of aphids and thrips, respectively, were counted together. For *M. persicae* the percentage of the leaf area covered by honeydew and for *F. occidentalis* the area covered by feeding and feces spots, respectively, were estimated per leaf. Leaves of the control treatment were additionally checked for symptoms of pest damage and the presence of pests but were free of both.

### Greenhouse experiment

#### General setup

The same variety of bell pepper plants used in the experiment under controlled conditions was cultivated in a soil greenhouse in 2022. The plants were sown during calendar week 14, cultivated in 100 mm pots and transferred to the greenhouse during calendar week 18. Three beds with widths of 500 mm were installed. Each bed contained a row of 20 plants. The planting distance within each row was 500 mm. Irrigation was performed by spraying at ground level. Fertilizer was applied every second week with Ferty 2 Mega/0.5‰ (Planta Düngemittel GmbH, Germany).

**Fig. 1:**
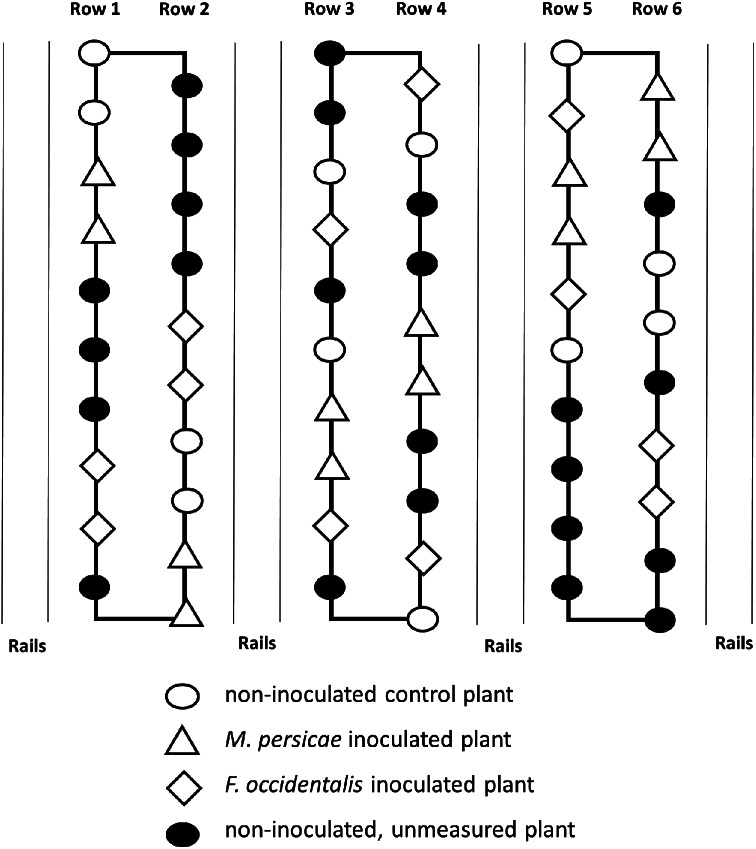
Greenhouse test setup. Test setup with three rows in a soil greenhouse in 2022. The thin lines indicate the rails on which the mobile HSI measurement platform was driven. The marked rows of symbols represent the individual plants, and the symbol shapes represent the respective plant treatments

Twelve individual plants were inoculated in calendar week 33, with 20 adult *M. persicae* or 40 adult *F. occidentalis*. Insects were distributed on the upper and middle leaves. These plants were distributed evenly, with two plants per treatment in each row. Another 12 plants were used as uninfested controls. During the cultivation period in the soil greenhouse, the plants that were measured with the his method were subsequently covered with a net with a mesh size of 0.35 mm (BIOTHRIPS 346, MDB Texinov, France) during the full cultivation period. Additionally, the plants were inspected weekly throughout the growing season, and unintended pest infestations were controlled by hand. As unintended pest occurrence was noticed and could not always be impeded during these inspections, some of the UC plants were replaced with remaining unused plants, which were then covered by nets accordingly. The final positions of the plants measured with the HSI method are shown in Fig. 1.

When the treatments were covered, additional beneficial insects were introduced into the greenhouse to limit the natural occurrence of target pests. A one-unit parasitic wasp solution (“VerdaProtect”, from Katz Biotech AG, Baruth, Germany), which is recommended for aphids in a 200 m² greenhouse area and contains a mixture of *Praon volucre*,* Aphidius matricariae*,* Aphidius colemani*,* Aphidius ervi*,* Aphelinus abdominalis* and *Ephedrus cerasicola*, was applied. The mixture was distributed evenly to the open-growth plants under the nets of the UC and FO treatments. The predatory mite *Amblyseius cucumeris* was used to control thrips, with 10,000 mites distributed among the open-grown plants and under the nets of the UC and MP treatments. The wasps and mites were evenly distributed on crops, including areas under nets, every 2 weeks. The application of beneficial insects ended two weeks before the inoculation of the FO and MP treatments, which took place two weeks before the first HSI measurement.

#### Construction of the mobile measurement platform

**Fig. 2:**
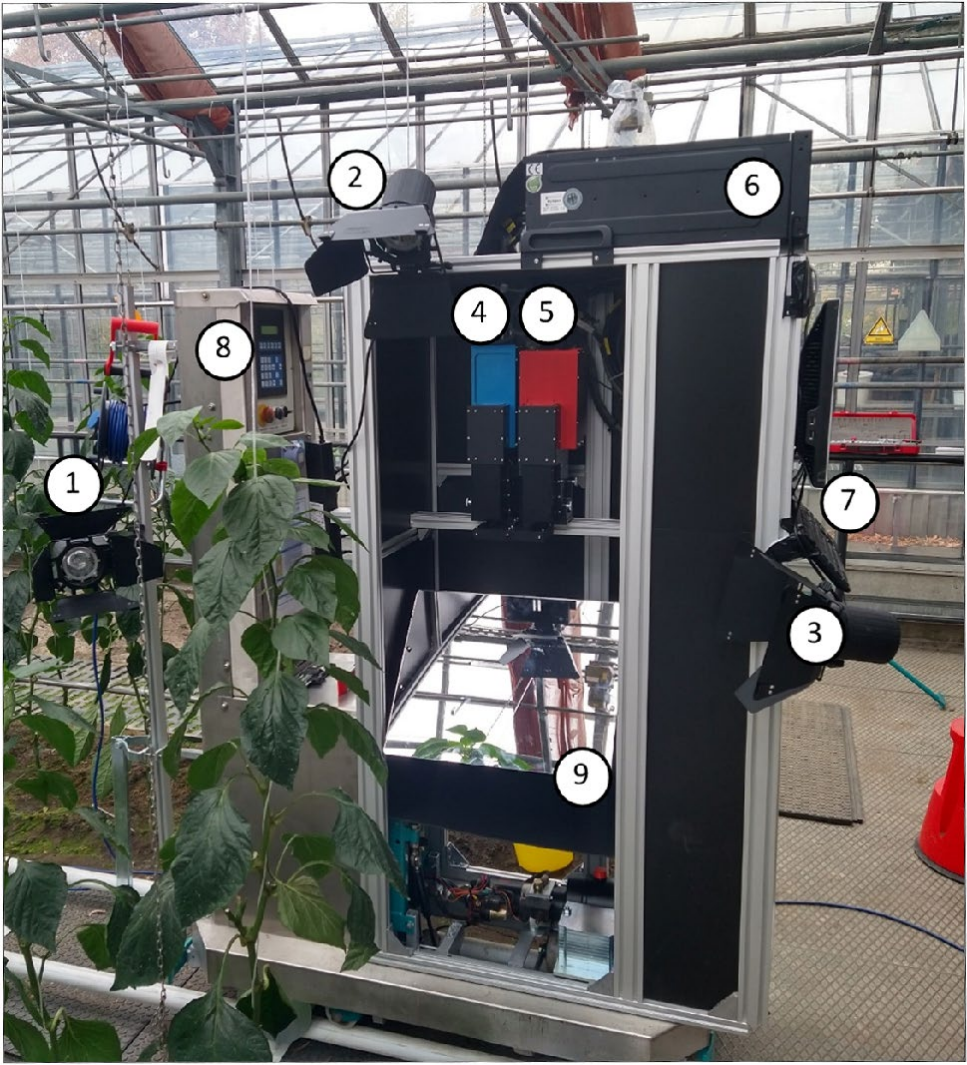
HSI measurement platform. Self-constructed mobile HSI measurement platform on rails in the experimental greenhouse with bell peppers grown in double rows. The stand consists of 300 W halogen spotlights (1–3), two hyperspectral cameras, a Hyspex Vis-NIR at 400,950 nm (4), a Hyspex NIR-SWIR at 950 bis 2500 nm (5), the control computer (6), the input device (7), the spraying robot control (8) and an angle mirror (9)

The hyperspectral measurements in the soil greenhouse experiment were carried out via a custom-constructed mobile HSI measurement platform. The basis for the stand was the spray robot platform “Meto” (Berg Hortimotive, De Lier, Netherland), which has a length of 1.70 m and a weight of 330 kg. The Meto platform can be automatically moved on heat rails at different speeds and is commonly used in soilless cultivation systems. With aluminum profiles from the company Mejo Metall Josten GmbH & Co., KG (Düsseldorf, Germany), a measurement structure was constructed on the platform. The HSI cameras, an angle mirror, two full-spectrum lamps of 400 W (Osram, Munich, Germany) and a computer for camera control were attached to the platform (Fig. 2). An external cable provided the power supply, and a mobile rail system was placed on the ground to operate the platform.

#### Hyperspectral image measurements in the greenhouse

HSI images were taken continuously for two weeks in September via the constructed mobile hyperspectral measurement platform (Fig. 2). All the plants in each treatment group were measured on three dates: Sept. 11, Sept. 15 and Sept. 23 in 2022. As an example, measurements during the daytime with natural light and no shading are presented. Other scenarios, such as measurements obtain at night with and without artificial light, were also explored, but did not perform better and are not shown in this paper.

#### Pest monitoring in the greenhouse

During the cultivation phase in 2022, bell pepper plants were inspected every week, such as via the use of yellow sticky traps and visual monitoring. Pests were monitored one day after HSI measurement on the inoculated and control plants. Three height ranges were defined, one representing the measurement range of the spectral camera (0.60–1.20 m), one representing the area above this range (1.20 m from the shoot tip) and one below this range (0.60 m). The classification of pests was carried out on the basis of the following categories: 0 = no insects, 1 = 1–5, 2 = 6–10, 3 = 11–15, 4 = 16–20, 5 = 21–30, 6 = 31–50, 7 = 51–70, 8 = 71–100, and 9 = more than 100 insects.

### Implementation of the evaluation algorithm

#### Dataset for hyperspectral image analysis

To develop an algorithm for predicting pest symptoms in bell peppers, the focus was on specific detection and differentiation among *M. persicae*, *F. occidentalis* and the noninfested control group. For prediction, the XGBoost framework (decision trees) in Python was used.

To train the prediction algorithms, images were acquired in a controlled laboratory environment with strict settings for light, distance and leaf placement, as described in Chap. 2.1.

#### Data preprocessing and image labeling

The different training methods of the prediction algorithms for two- and three-class classification were performed. Different pest infestation cases were used with the three classes UC, MP and FO. The images were labeled with the specific features in their filenames.

#### Leaf segmentation

**Fig. 3:**
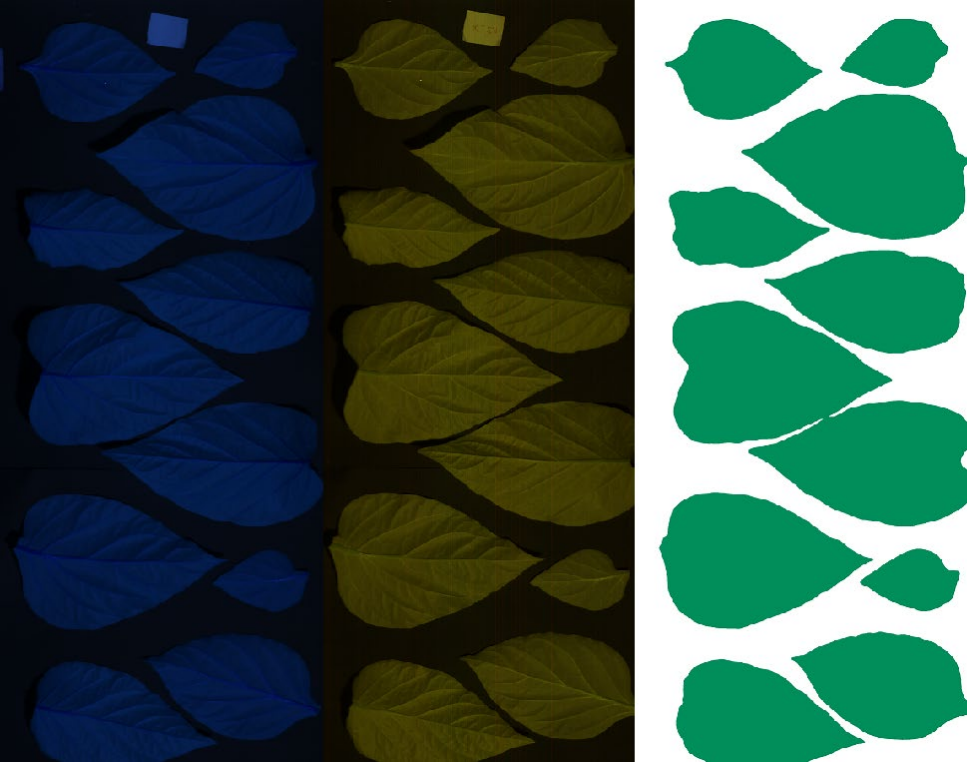
Visualization and segmentation of images. Example of a 3-channel visualization of an SWIR image (left) and a VNIR image (middle), together with the resulting leaf segmentation mask created via deep learning (right)

To improve the algorithm performance, it is helpful to remove the image background and retain only the leaf area for analysis (Fig. 3). As a first step in leaf segmentation, a 3-channel PNG image was created for each of the hyperspectral images. For each of the VNIR and SWIR images, the 20th, 120th and 180th bands of the data file were selected. These corresponded to the wavelengths of 464, 780 and 972 nm, respectively, for the VNIR camera and the 1058 nm1605 and 1930 nm wavelength bands for the SWIR camera. The selection of wavelengths was performed experimentally with the aim of creating high-contrast visualizations of the data, optimized for manual inspection and segmentation annotation.

For actual leaf segmentation, deep learning technology was used, namely, the Deeplab3 algorithm (Fig. 3). Deeplab3 is one of the best-performing image segmentation algorithms for the Pascal VOC dataset and is often used as a benchmark for segmentation quality.

During training, the neural network weights were fine-tuned via the training script provided by Google Research. The neural network was used to analyze image patches of size 5122 pixels. To train the network, randomly cropped labeled image patches from the training dataset were used. 80% of the labeled data were used for training, and 20% were used to evaluate the segmentation quality (the test data). Each dataset was trained until the training error converged. Using the test data, it was verified that no overtraining occurred. To train the leaf segmentation algorithm, a dataset consisting of 80 of the created 3-channel images was used.

#### Features and tiles

**Fig. 4:**
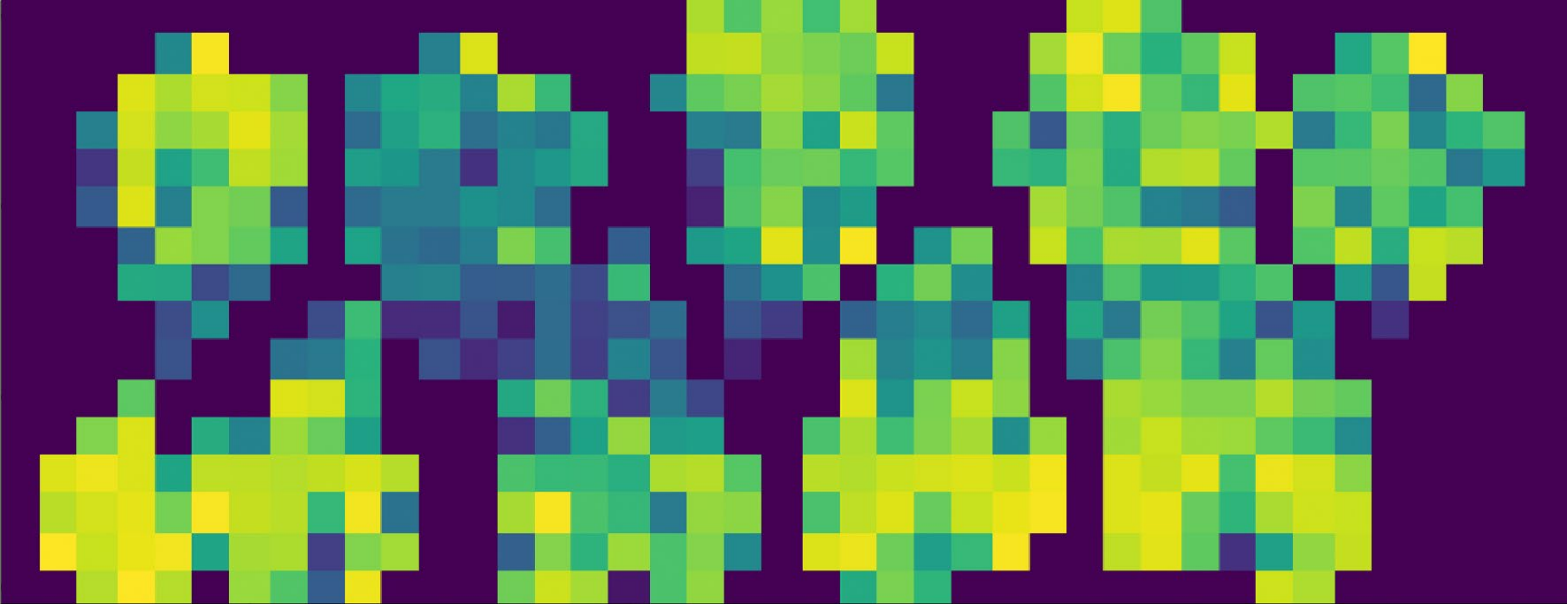
Visualization of tiles. Visualization of the different mean values for a single wavelength in a tiled image

To increase the number of data points, each image was divided into several tiles (Fig. 4). With respect to tile size, 25 × 25 pixels were used for the SWIR images, and 111 × 111 pixels were used for the VNIR images. The size ratio between the SWIR and VNIR tiles was related to the size ratio of the complete images. This supported the convenient combination of both image types for processing with one prediction algorithm. As features for the training of the gradient boosted trees, the mean values for each tile and wavelength were calculated. Adding the standard deviation of each wavelength within each tile as an additional feature did not have a positive effect on the training results.

#### Fitting and hyperparameters

For training and prediction, the XGBoost framework, which is available in Python, is an algorithm based on gradient-boosted trees. Gradient-boosted trees are state-of-the-art submodels in terms of processing speed and are highly popular in the machine learning community. Many parameters of this algorithm can be modified, such as the learning rate, the maximum depth of a tree or the number of trees. To find appropriate hyperparameters for this regression problem, a grid search was conducted, and the most appropriate hyperparameters yielding the lowest RMSE were used for further evaluation. The parameters obtained via grid searches that typically yield the lowest prediction error are 0.05 for the learning rate, 10 for the maximum tree depth and approximately 100 for the number of trees.

To assess the accuracy of detection, 70% of the data were used for training, and 30% were used to evaluate the prediction quality (the test data). The dataset was split into training and testing sets, which were randomized with an equal number of images per class. A broad variety of 2-class and 3-class training methods for both image types were implemented.

After the trained algorithms were evaluated with separate images of the top and bottom sides of leaves, a test was performed with images of whole plants. To perform a robust test, only images of whole plants whose cut leaves were not used for training of the algorithm were used.

#### Selection of important wavelengths

A benefit of using gradient boosting is that after the boosted trees are constructed, it is relatively straightforward to retrieve importance scores for each attribute. In general, the importance score indicates how useful or valuable each feature is in the construction of the boosted decision trees within the model. The more an attribute is used to make key decisions in decision trees, the greater its relative importance. Functions integrated into XGBoost were used in a three-class prediction algorithm to assess the importance of features, and important wavelengths were identified.

#### Application of the algorithm to the greenhouse dataset

For use in a greenhouse environment, the algorithm trained with images of the top and bottom sides of leaves was tested regarding the distinction of the three classes of pest infestation. By using images of both sides of leaves in the training data, a high variety of image content was ensured while also significantly increasing the size of the training dataset. Notably, this approach was used to make the algorithm more robust to variable orientations of leaves in later analyses with images from the greenhouse environment. After one image was taken per row of plants, the horizontal pixel range of the test plants was labeled manually. After images were analyzed via the three-class prediction algorithm with combined camera data, a percentage was calculated per plant regarding the number of tiles for which the algorithm calculated a probability of > 80 for an infestation of *M. persicae* or *F. occidentalis*. Additionally, the overall trends were analyzed. The percentage of tiles with a predicted > 80% infestation probability per plant was obtained and compared to the pest information manually observed for the same plant.

## Results

### Experiment under controlled conditions

For FO, an average of 1.8 ± 1.25 [mean ± SD] *F. occidentalis* individuals per leaf were found on each of 887 leaves, and 9.56 ± 4.15% [mean ± SD] of the leaf area displayed feeding symptoms and/or feces. In the case of MP, an average of 46.55 ± 62.55 (mean ± SD) *M. persicae* individuals per leaf were found on each of the 902 leaves, and 90.62 ± 9.19 (mean ± SD) of the leaf area was covered by honeydew. Leaves from control plants were free of insects and symptoms.

The predictions for both the MP and FO treatments compared with those for the control group UC are shown in Table 2. Generally, high prediction success was achieved, with the correct classification greater than 80% for precision and recall when a test set of single leaves was used and approximately 70% when full plants were used in the test set.

**Table 2:** Predictions under controlled conditions

	Precision	Recall	F1-score	Support
Single leaves, two classes (M. persicae, control group)				
Control group	0.90	0.81	0.85	32
*M. persicae*	0.83	0.91	0.87	32
Accuracy			0.86	64
Macro avg	0.86	0.86	0.86	64
Weighted avg	0.86	0.86	0.86	64
***Single leaves***,*** two classes (F. occidentalis***,*** control group)***				
Control group	0.86	1.00	0.92	30
*F. occidentalis*	1.00	0.83	0.91	30
Accuracy			0.92	60
Macro avg	0.93	0.92	0.92	60
Weighted avg	0.93	0.92	0.92	60
***Single leaves***,*** three classes (M. persicae***,*** F. occidentalis***,*** control group)***				
Control group	0.90	0.84	0.87	31
*F. occidentalis*	0.94	0.97	0.95	31
*M. persicae*	0.91	0.94	0.92	31
Accuracy			0.91	93
Macro avg	0.91	0.91	0.91	93
Weighted avg	0.91	0.91	0.91	93
***Full plant***,*** three classes (M. persicae***,*** F. occidentalis***,*** control group)***				
Control group	0.68	0.74	0.71	31
*M. persicae*	0.70	0.61	0.66	31
*F. occidentalis*	0.66	0.68	0.67	31
Accuracy			0.68	93
Macro avg	0.68	0.67	0.68	93

Prediction under controlled conditions for single leaves and full plants for the two classes of *M. persicae* or *F. occidentalis* versus the control group and all three classes were performed. The analyses included data from the SWIR and VNIR cameras as well as images of the top and bottom sides of the measured leaves in the training dataset. Algorithms were then applied to test datasets with images of leaves and full plants.

**Fig. 5:**
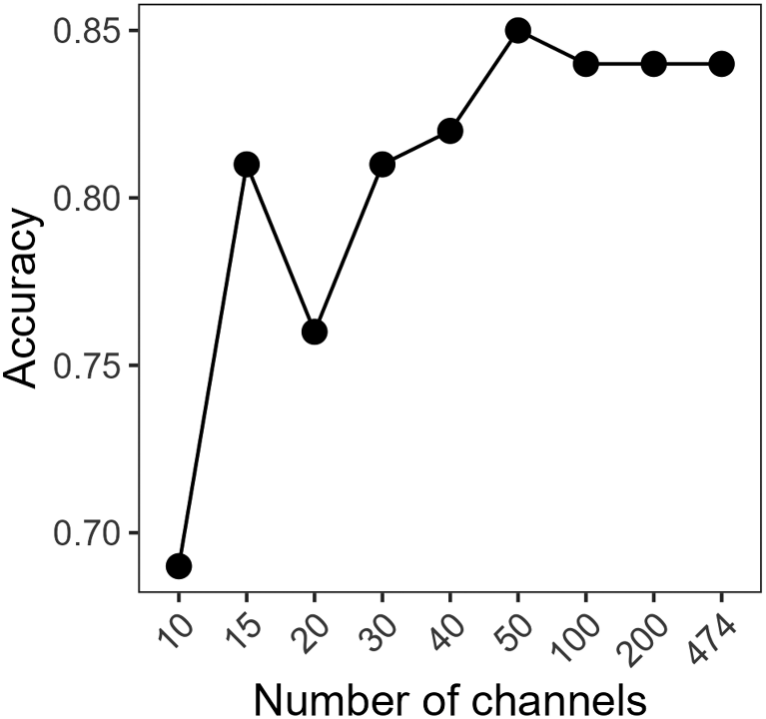
Influence of wavelength number on accuracy. Changes in the accuracy of the three-class prediction algorithm under controlled conditions when reducing the number of wavelength channels used. Analyses were carried out via the XGBoost integrated functions with images of the top and bottom sides of leaves

On the basis of the results of XGBoost, the 15 (**10**) most significant bandwidths for the prediction of the three classes UC, FO and MP were **455**, 502, **720**, **955**, **980**, **1340**, **1356**, **1370**, **1518**, 1525, 1532, **1833**, 1840, 1496, and **2500** nm. Development of the algorithm’s performance is shown in Fig. 5.

### Greenhouse test

**Fig. 6:**
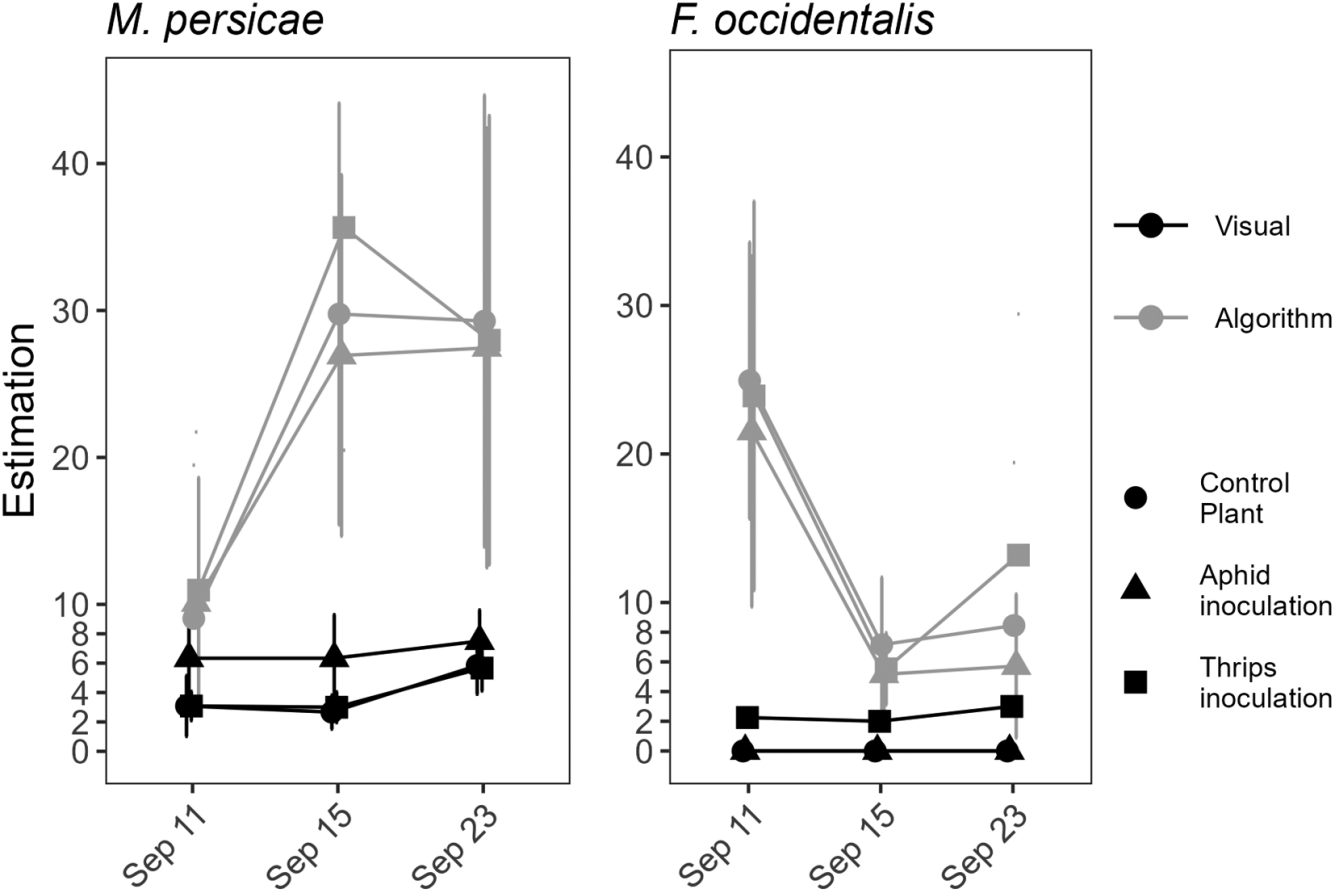
Comparison of visual and algorithm results under greenhouse conditions. Visual estimates based on insect count categories from 0 (no insect) to 9 (> 100 insects) (gray) and algorithm estimates given as the percentage tiles with > 80% predicted infestation probability (black). The different treatments are shown separately, as are the infestations of *M. persicae* and *F. occidentalis* in each treatment

During visual monitoring, none of the net-covered treatments remained free of aphids during the trial. At the first sampling date, Sept. 11, *M. persicae* was present on all the plants in all the treatment groups, including those in the UC and FO groups (Fig. 6). For *F. occidentalis*, the numbers were generally low, and the plants in the UC and MP treatments remained free of thrips. However, neither the numerical differences in pest densities between the treatments at the daily scale nor the trends in the densities over time were similar to the predictions of the algorithm applied.

## Discussion

Hyperspectral imaging has high potential for the detection of plant quality parameters and plant stress. However, most studies have not tested the possibility of distinguishing among different stress factors or, as in the present study, the presence of different insects or pests. For example, Zhao et al. [11] reported that the detection of *M. persicae* infestation on Chinese cabbage was possible, and Mohite et al. [12] were able to discriminate low-level thrips infestations on capsicum leaves in relation to healthy leaves. Peignier et al. [7] reported that aphid species on plants can be distinguished via HSI. In the present study, *M. persicae* and *F. occidentalis* infestations were distinguished from each other and from an uninfested control. Furthermore, the developed algorithm proved to be robust to differences in the presentation of leaves to the camera, as long as the light and background conditions remained the same. This was demonstrated by applying the algorithm developed on single, horizontally presented leaves without overlap to full single plants that included other organs, such as stems, and displayed leaf overlap and various leaf positions, angles and distances to the camera. Evaluating the performance of the algorithm on the basis of single plants can be seen as an intermediate step between applications under controlled conditions and greenhouse conditions. The overall performance of the algorithm decreased from approximately 90% to approximately 70% when it was applied to full single plants. However, the bell pepper plants used for this study were chosen because of their simple leaf structure and smooth leaf surface, making them easy to study with machine learning algorithms. It remains to be tested whether such an algorithm would perform similarly if applied to more complex leaves, such as leaves covered with trichomes, as typically found in solanaceous greenhouse vegetable crops. Additionally, the feeding habits and damage types were quite different for the insects tested in this study. Thrips feed on single plant cells, leaving whitish spots on plants and dark fecal droppings on leaves. Aphids, on the other hand, feed on phloem sap without causing major damage to plant tissues [13], but they produce honeydew that covers the surrounding leaves. This coverage is shiny due to the high sugar content and may be covered by sooty mold later. These differences likely facilitated discrimination via HSI, whereas distinguishing several phloem feeders may be more challenging.

To test the robustness of the developed algorithm on the basis of a prototype automated system under greenhouse conditions, the described mobile hyperspectral measurement platform was constructed and applied to assess bell peppers grown under practical conditions. Unfortunately, the detection of pest infestations failed under these conditions. There are several possible reasons for this result, such as technical, environmental and human-related issues. Technically, it was not possible to fine-tune the speed of the prototype to the speed of the linescan setup used under controlled conditions, resulting in slightly compressed images. Additionally, the range of the distance to the leaves was highly variable in this case given the more voluminous structure of the larger plants in the greenhouse. Moreover, the environmental conditions, especially light conditions, were not controlled under greenhouse conditions. Although installed lamps comparable to those in the controlled setup were used on the measurement platform, ambient light likely influenced the outcome of the prediction process. However, this problem was also not overcome by performing night measures because artificial light was not bright enough to obtain effective measurements (data not shown). Additionally, the age of the plant could have played a role in the detection result, with the greenhouse plants being older than the ones in the controlled setup owing to limitations in the cage and setup size under controlled conditions. In addition, comparing prediction outputs with visual monitoring data is not trivial. The first output of the algorithm was a heatmap, which shows the analyzed image tiles. For comparison, a rating was extracted by calculating the percentage of tiles with a high probability of pest damage or presence compared with the overall number of tiles. However, as labeling of the plant width in images could only be performed manually, different shares of tiles without plant tissue on them may have been included in the analysis because leaf segmentation, as in the controlled setup, was not possible. Leaf segmentation was attempted by using black cardboard as the background, but this did not enhance the results of the analyses (data not shown). Additionally, the visually monitored plant segment was generally the same as that in images, but some variability likely occurred. Moreover, treatments in the greenhouse environment could not be kept free of unintended pests, such as aphids. As a result, the algorithm, which was trained using images of leaves with only one pest species, struggled to detect multiple infestations under greenhouse conditions. Therefore, it cannot be clearly confirmed that the algorithm would have failed if multiple infestations in the greenhouse could have been controlled. However, in practice, multiple infestations are common, and the robustness of algorithms to these scenarios is needed for practical use. As the inclusion of multiple infections would greatly increase the size of necessary training datasets and computing power, development of HSI for practical conditions is challenging. The application of algorithms trained under controlled conditions in single-pest scenarios to multiple-infestation scenarios should be tested in the future to assess robustness to these variations.

For further development of the detection system, reducing the cost of the hardware system is necessary. In the current setup, the hyperspectral cameras are the most expensive components. For practical application, it could be necessary to switch to a cheaper system, such as one with industrial multispectral cameras. Such multispectral cameras provide information at significantly fewer wavelengths. In this study, the use of information from 15 wavelengths reduced the accuracy only slightly compared with the use of information from 200 wavelengths. However, most of the important wavelengths were monitored by the SWIR camera only (10) and not the VNIR camera (3); two wavelengths were detected by both cameras. Given that cameras for monitoring the SWIR spectrum are expensive, cost constraints may limit the applicability of the proposed technique, at least for the given crop-pest combination.

In this study, the focus was not on early detection based on possible changes within the plant in response to a pest attack; rather, the pest population had two weeks to damage the plant and develop different developmental stages. Notably, the authors saw major value in the automated detection of pest symptoms using plant images obtained via automated monitoring. That is, pictures can be taken and analyzed automatically in high numbers throughout large-scale, specialized greenhouses. These greenhouses already have highly developed infrastructure for the deployment of autonomous tools within the greenhouse, for example, spray robots that navigate automatically between rows moving on installed heat pipes or other structures. This infrastructure could be used without high adaptation costs. As manual monitoring is highly time and labor intensive, it is seldom carried out in depth throughout the growing season [14, 15]. Automated monitoring could therefore markedly increase accuracy in time and space, regardless of whether detection is possible earlier than human observations are effective. Nevertheless, this HSI approach should be considered in a later step. However, as good results using images in the visible spectrum or RGB images have been previously obtained [16], the benefits of HSI alone or in combination with other methods should be evaluated.

Overall, the transition from image analyses under controlled conditions to analyses in the greenhouse environment was challenging in this study. Many studies have shown the high potential of HSI for the detection of infested plants from pest-free controls under very controlled conditions [11, 17–21], but few studies have successfully shown the same results under real-world conditions [4, 22] or that the symptoms of different pest organisms can be distinguished under real-life conditions [23, 24]. Given the greenhouse conditions in this study, the authors feel that a prediction algorithm using HSI should be developed under conditions that are closer to those in practice in the future, thus allowing the algorithm to be applied under real-life conditions, such as in greenhouses or in the field. Also, in this context, a significant expansion of the training dataset is necessary to improve generalization of the algorithms. On the technical side, potential is seen in improving the lighting conditions. Additionally, from our perspective, the use of distance data, which can be obtained, for example, through an additional stereoscopic RGB camera, as an additional feature during the training of the algorithms, can help to improve the application of the algorithms in the greenhouse environment.

## Conclusion

In this study, hyperspectral imaging over a wide spectrum from 400 to 2500 nm enabled noninvasive determination and the distinction of healthy plants and plants infested with *M. persicae* and *F. occidentalis* on bell peppers. Important wavelengths were associated with the infestation patterns of different insects. The method was successful for single-cropped leaves and, with some loss in accuracy, for small whole plants under controlled conditions. However, applications under greenhouse conditions did not result in a good fit with the results of manual monitoring. This study shows the potential of using hyperspectral images for the detection of sucking pests on bell peppers, even when multispectral camera systems are used. To generalize our approach, it would be beneficial to include additional pests or stress factors in subsequent studies. The application of automated platforms similar to the one tested in this study is possible, but for successful pest symptom detection under greenhouse conditions, algorithms based on real-world conditions should be developed. Overall, the transition from images analyses under controlled conditions to analyses in the greenhouse environment was too complex in this study. It remains to be determined if this is a specific problem for the given plant–pest combination considered in our experiments or if this is a general finding.

## Data Availability

The datasets used and analysed during the current study are available from the corresponding author on reasonable request.

## References

[1] Stenberg JA. A conceptual Framework for Integrated Pest Management. Trends Plant Sci. 2017;22:759–69. 10.1016/j.tplants.2017.06.010. 28687452 10.1016/j.tplants.2017.06.010

[2] Lowe A, Harrison N, French AP. Hyperspectral image analysis techniques for the detection and classification of the early onset of plant disease and stress. Plant Methods. 2017;13:80. 10.1186/s13007-017-0233-z. 29051772 10.1186/s13007-017-0233-z PMC5634902

[3] Mahlein A-K, Steiner U, Dehne H-W, Oerke E-C. Spectral signatures of sugar beet leaves for the detection and differentiation of diseases. Precision Agric. 2010;11:413–31. 10.1007/s11119-010-9180-7.

[4] Nguyen HD, Nansen C. Hyperspectral remote sensing to detect leafminer-induced stress in bok choy and spinach according to fertilizer regime and timing. Pest Manag Sci. 2020;76:2208–16. 10.1002/ps.5758. 31970888 10.1002/ps.5758 PMC7317203

[5] Ahmad MN, Shariff ARM, Moslim R. Monitoring insect pest infestation via different spectroscopic techniques. Appl Spectrosc Rev. 2018;53:836–53. 10.1080/05704928.2018.1445094.

[6] Lima MCF, Damascena de Almeida Leandro ME, Valero C, Pereira Coronel LC, Gonçalves Bazzo CO. Automatic Detect Monit Insect Pests—A Rev Agric. 2020;10:161. 10.3390/agriculture10050161.

[7] Peignier S, Lacotte V, Duport M-G, Baa-Puyoulet P, Simon J-C, Calevro F, et al. Detection of aphids on hyperspectral images using one-class SVM and Laplacian of gaussians. Remote Sens (Basel). 2023;15:2103. 10.3390/rs15082103.

[8] Ragsdale DW, McCornack BP, Venette RC, Potter BD, Macrae IV, Hodgson EW, et al. Economic threshold for soybean aphid (Hemiptera: Aphididae). J Econ Entomol. 2007;100:1258–67. 10.1093/jee/100.4.1258. 17849878 10.1603/0022-0493(2007)100[1258:ETFSAH]2.0.CO;2

[9] Li L, Xie S, Ning J, Chen Q, Zhang Z. Evaluating green tea quality based on multisensor data fusion combining hyperspectral imaging and olfactory visualization systems. J Sci Food Agric. 2019;99:1787–94. 10.1002/jsfa.9371. 30226640 10.1002/jsfa.9371

[10] Li S, Song W, Fang L, Chen Y, Ghamisi P, Benediktsson JA. Deep learning for hyperspectral image classification: an overview. IEEE Trans Geosci Remote Sens. 2019;57:6690–709. 10.1109/TGRS.2019.2907932.

[11] Zhao Y, Yu K, Feng C, Cen H, He Y. Early detection of aphid (Myzus persicae) Infestation on Chinese Cabbage by Hyperspectral Imaging and feature extraction. Trans ASABE. 2017;60:1045–51. 10.13031/trans.11886.

[12] Mohite J, Gauns A, Twarakavi N, Pappula S. In: Thomasson JA, McKee M, Moorhead RJ, editors. Evaluating the capabilities of Sentinel-2 and Tetracam RGB + 3 for multi-temporal detection of thrips on capsicum. Volume 106640U. SPIE; 2018. 10.1117/12.2305358.

[13] Tjallingii WF, Esch TH. Fine structure of aphid stylet routes in plant tissues in correlation with EPG signals. Physiol Entomol. 1993;18:317–28. 10.1111/j.1365-3032.1993.tb00604.x.

[14] Steiner MY, Spohr LJ, Barchia I, Goodwin S. Rapid estimation of numbers of whiteflies (Hemiptera: Aleurodidae) and thrips (Thysanoptera: Thripidae) on sticky traps. Aust J Entomol. 1999;38:367–72. 10.1046/j.1440-6055.1999.00114.x.

[15] Cullen E, Zalom F, Flint M, Zilbert E. Quantifying trade-offs between pest sampling time and precision in commercial IPM sampling programs. Agric Syst. 2000;66:99–113. 10.1016/S0308-521X(00)00038-X.

[16] Fuentes A, Yoon S, Park DS. Deep learning-based techniques for plant diseases recognition in real-field scenarios. In: Blanc-Talon J, Delmas P, Philips W, Popescu D, Scheunders P, editors. Advanced concepts for Intelligent Vision systems. Cham: Springer International Publishing; 2020. pp. 3–14. 10.1007/978-3-030-40605-9_1.

[17] Xie C, Yang C, He Y. Hyperspectral imaging for classification of healthy and gray mold diseased tomato leaves with different infection severities. Comput Electron Agric. 2017;135:154–62. 10.1016/j.compag.2016.12.015.

[18] Zhou R-Q, Jin J-J, Li Q-M, Su Z-Z, Yu X-J, Tang Y, et al. Early detection of Magnaporthe oryzae-infected Barley leaves and Lesion visualization based on hyperspectral imaging. Front Plant Sci. 2018;9:1962. 10.3389/fpls.2018.01962. 30697221 10.3389/fpls.2018.01962 PMC6341029

[19] Xie C, Shao Y, Li X, He Y. Detection of early blight and late blight diseases on tomato leaves using hyperspectral imaging. Sci Rep. 2015;5:16564. 10.1038/srep16564. 26572857 10.1038/srep16564 PMC4647840

[20] Barreto A, Paulus S, Varrelmann M, Mahlein A-K. Hyperspectral imaging of symptoms induced by Rhizoctonia solani in sugar beet: comparison of input data and different machine learning algorithms. J Plant Dis Prot. 2020;127:441–51. 10.1007/s41348-020-00344-8.

[21] Kong W, Zhang C, Huang W, Liu F, He Y. Application of hyperspectral imaging to detect sclerotinia sclerotiorum on oilseed rape stems. Sens (Basel). 2018. 10.3390/s18010123. 10.3390/s18010123 PMC5796448 29300315

[22] Thomas S, Kuska MT, Bohnenkamp D, Brugger A, Alisaac E, Wahabzada M, et al. Benefits of hyperspectral imaging for plant disease detection and plant protection: a technical perspective. J Plant Dis Prot. 2018;125:5–20. 10.1007/s41348-017-0124-6.

[23] Hillnhütter C, Mahlein A-K, Sikora RA, Oerke E-C. Remote sensing to detect plant stress induced by Heterodera schachtii and Rhizoctonia solani in sugar beet fields. Field Crops Res. 2011;122:70–7. 10.1016/j.fcr.2011.02.007.

[24] Hillnhütter C, Mahlein A-K, Sikora RA, Oerke E-C. Use of imaging spectroscopy to discriminate symptoms caused by Heterodera schachtii and Rhizoctonia solani on sugar beet. Precision Agric. 2012;13:17–32. 10.1007/s11119-011-9237-2.

